# Surgical Management of Urinothorax After Percutaneous Nephrolithotomy

**DOI:** 10.1016/j.atssr.2023.11.018

**Published:** 2023-12-09

**Authors:** Johanna Lou, Dominick M. Scalia, Yazid Ghanem, Kenji Minakata, David Shersher

**Affiliations:** 1 Department of Surgery, Cooper University Hospital, Camden, New Jersey; 2 Cooper Medical School of Rowan University, Camden, New Jersey; 3 Division of Cardiac Surgery, Department of Surgery, Cooper University Hospital, Camden, New Jersey; 4 Division of Thoracic Surgery, Department of Surgery, Cooper University Hospital, Camden, New Jersey

## Abstract

Urinothorax is a rare form of pleural effusion that can occur after percutaneous instrumentation of the genitourinary tract. We report on the surgical management of a 75-year-old man with a past surgical history of 3-hole esophagectomy who was afflicted with a loculated urinothorax complicated by sepsis after a supracostal percutaneous nephrolithotomy. We demonstrate the efficacy of pleurodesis and decortication in the management of complicated urinothorax that is refractory to medical management.

Urinothorax, considered a rare form of pleural effusion, is the accumulation of urine in the pleural space. It arises from a variety of causes that can be broadly classified as obstructive uropathies or traumatic.^1^^,^^2^ Most cases are iatrogenic after instrumentation of the genitourinary tract, such as nephrostomy tube placement, percutaneous nephrolithotomy (PCNL), double J-stent, and lithotripsy.^2^ Diagnosis of urinothorax requires a high index of suspicion and careful consideration of the clinical context. One hallmark finding is a pleural fluid to serum creatinine ratio of >1, especially in the context of recent urologic intervention or obstruction.^3^

The mainstay for treatment of urinothorax is correction of the underlying uropathy or trauma, with or without thoracic drainage.^1^^,^^2^ Thoracic surgical approaches to urinothorax, including decortication and pleurodesis, are rarely used because decompressing the urinary system alone usually leads to rapid resolution of symptoms.^1^^,^^2^ In cases of true renopulmonary fistula or when there are advanced pleural sequelae (such as loculated effusion), thoracic interventions can be effective. Here we report the case of a 75-year-old man who underwent a video-assisted thoracoscopic decortication and talc pleurodesis for a loculated right-sided urinothorax after PCNL, nephrostomy tube removal, and placement of a double J-stent.

A 75-year-old man with a distant past medical history of 3-hole minimally invasive esophagectomy, interstitial lung disease secondary to asbestos exposure, benign prostatic hyperplasia, right renal cyst, recurrent right nephrolithiasis, and chronic kidney disease presented with a right staghorn calculus, for which the interventional radiologist placed a right percutaneous nephroureteral tube (PCN). The urologist next performed a right PCNL through an access point above the 12th rib under fluoroscopic guidance. A right-sided pneumothorax developed, and the patient required a small-bore pleural catheter placement. He recovered and was discharged from the hospital 2 days later. One week later, the patient returned for a second-look nephrostogram and underwent repeated right PCNL for residual stones with internal drainage by double J-stents. Immediately after the second urologic procedure, he became febrile, and a right pleural effusion developed. A therapeutic thoracentesis was performed, with resolution of symptoms and discharge. Results of culture and cytology of the pleural fluid were negative for organisms and malignant cells, although chemical studies were not performed.

The patient presented again to the emergency department 5 days later with dyspnea and right-sided pleuritic chest pain. Chest imaging redemonstrated a large right pleural effusion (Figure 1). Blood chemistry studies revealed that the patient’s serum creatinine was 3.89 mg/dL, above his baseline. A small-bore pleural catheter was placed into the right pleural space, which drained 2.5 L of serous fluid within the first 24 hours. Pleural fluid analysis showed a creatinine concentration of 11.80 mg/dL, lactate dehydrogenase level of 73 U/L, and protein level of 0.8 g/dL; culture was negative, and cytology was negative for malignant cells. The initial pleural fluid to serum creatinine ratio was approximately 3.03, consistent with a right-sided urinothorax.

**Figure 1:**
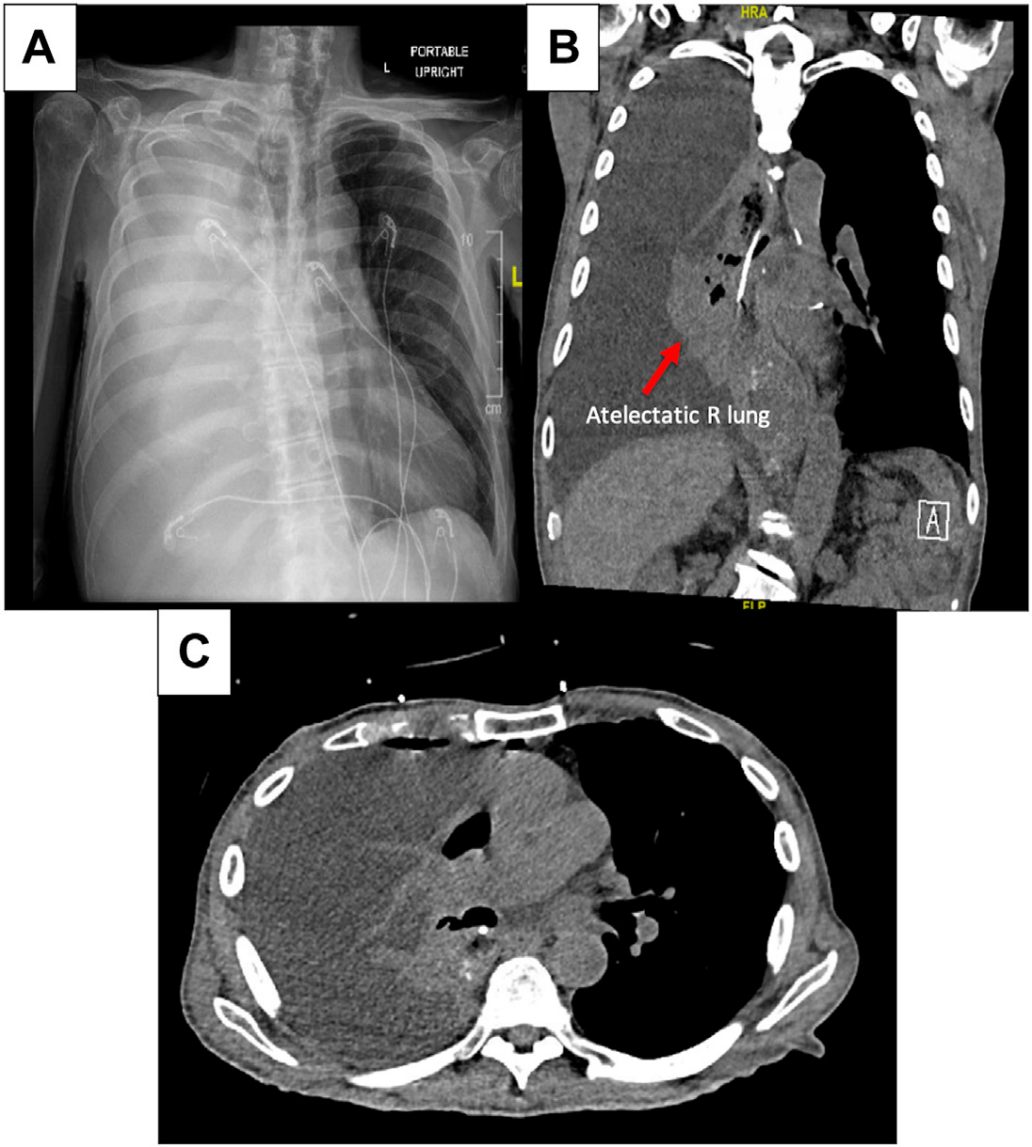
Initial chest imaging on presentation to the emergency department. (A) Chest film shows complete hemi-opacification of the right side of the chest. (B) Coronal computed tomography image shows nearly complete atelectasis of the right lung (arrow). (C) Axial computed tomography image shows right-sided hydropneumothorax. R, right.

The urinothorax was initially managed medically with intrapleural alteplase given through the indwelling pigtail, without complete resolution. A thoracic surgeon was later consulted for definitive surgical management as the patient had sepsis with concern for right-sided empyema. In preparation for thoracic surgery, intravenous broad-spectrum antibiotics were prescribed, and the interventional radiologist decompressed the urinary system with a new PCN. He subsequently underwent a video-assisted thoracoscopic decortication and talc pleurodesis. Access was challenging because of previous 3-hole minimally invasive esophagectomy, and great care was taken to avoid disruption of the gastric neoesophagus located in the posterior right side of the chest with success. Intraoperatively, there was a thick rind with multiloculated areas of urine and purulent fluid in the costophrenic recess. There was no visible defect in the diaphragm that contributed to the right-sided urinothorax, and 5 g of talc was used for chemical pleurodesis. Culture of the fluid was negative, likely sterilized after several days of antibiotics, and cytology was negative for malignant cells.

By postoperative day 3, all chest tubes were removed with complete resolution of right-sided urinothorax on chest radiography (Figure 2). The patient was eventually discharged with a PCN and double J-stent in place. The PCN was removed 1 month later, as preoperatively planned, without complications. The patient eventually followed up with the thoracic surgeon and urologist with complete renal and pulmonary recovery, without recurrence of nephrolithiasis.

**Figure 2:**
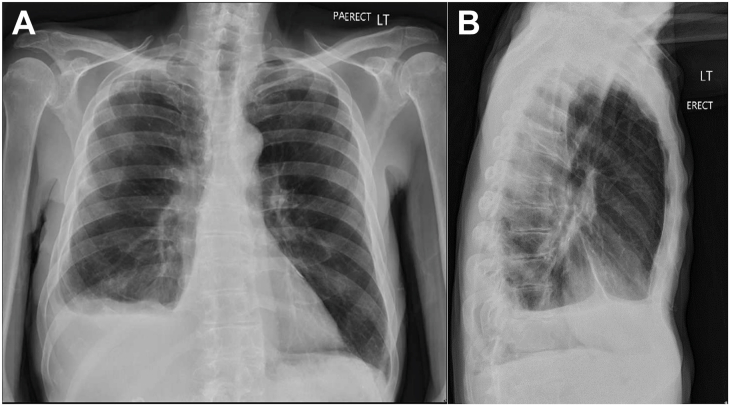
Two-view radiograph showing resolution of the right-sided urinothorax. (A) anterior view. (B) sagittal view.

## Comment

A urinothorax is a rare form of pleural effusion that has a variety of causes including injury during a supracostal approach to a PCNL,^4^^,^^5^ as demonstrated in our case. Supracostal access is typically avoided if possible; but when it is required, image-guided access with ultrasound or fluoroscopy can minimize potential intrathoracic complications. In our patient, fluoroscopy was used during PCNL, although employment of additional ultrasound guidance may have helped mitigate the risk of urinothorax. The hallmark biochemical finding of urinothorax is the pleural fluid to serum creatinine ratio of >1.^2^^,^^3^ Other characteristics, such as a pleural fluid protein level <1 mg/dL, color, and smell (serous fluid with an ammonia smell), help confirm the diagnosis.^2^

The mainstay for treatment of urinothorax is correction of the underlying mechanism, with the best outcomes seen when the underlying uropathy is corrected alone or in conjunction with thoracic drainage.^1^^,^^2^ When thoracic intervention is used alone, especially in cases of a missed pleural effusion, results are usually not satisfactory, and recurrence rates of pleural effusion are high.^1^ With adequate subdiaphragmatic urinary decompression tools, it is rare to need thoracic surgical interventions to definitively treat urinothorax.^1^^,^^2^ Thoracic surgery is used to prevent or to treat complications of long-standing effusion, such as multiloculated empyema, as we described in our case.^1^^,^^6^ Our case was unique, given previous history of 3-hole minimally invasive esophagectomy; there was concern that the index operation may have altered anatomy or caused fenestration in the right diaphragm that may have contributed to the refractory nature of the iatrogenic right-sided urinothorax. An attempt at nonoperative management of the right pleural space with decompression and alteplase instillation is reasonable, although in settings of mature renopulmonary fistula such as ours, this may have limited effect.^7^ This patient’s underlying interstitial lung disease likely also impaired full lung reexpansion and pleural lining apposition without operative intervention. The case highlights the complexity of urinothorax diagnosis in a patient who had undergone esophagectomy years earlier with malnutrition-induced refractory staghorn renal calculi; we showcase the use of a multidisciplinary team to adequately decompress the urinary system, to treat sepsis from right-sided empyema, and to provide successful minimally invasive right decortication and talc pleurodesis to drain and ultimately to obliterate the right pleural space. Only 1 other published case employed pleurodesis (mechanical) to effectively treat persistent urinothorax.^8^

Urinothorax is a rare subtype of pleural effusion that can lead to increased morbidity if it is not diagnosed and addressed promptly. In patients with recent percutaneous urologic interventions and a postintervention effusion, a high index of suspicion is necessary for a prompt diagnosis. In cases of patients who are refractory to medical management and pleural drainage, thoracic surgical approaches, including decortication and pleurodesis, are effective options to be added early as part of the armamentarium for treatment of complicated urinothorax.
